# The Natural History of Globus Pharyngeus

**DOI:** 10.1155/2010/159630

**Published:** 2010-12-27

**Authors:** E. C. Cashman, M. J. Donnelly

**Affiliations:** ^1^Dana-Farber Cancer Institute,Harvard Medical School, Longwood Avenue, Boston, MA 02115, USA; ^2^Department of Otolaryngology, Waterford Regional Hospital, Waterford, Ireland

## Abstract

Globus pharyngeus is a common disorder and accounts for 5% of all ENT referrals. *Objectives*. To evaluate the role of barium swallow and endoscopy in these patients, to ascertain the incidence, if any, of aerodigestive tract malignancy in this group and to assess the natural evolution of globus pharyngeus. *Materials and Methods*. Seventy-nine patients underwent barium swallow and rigid oesophagoscopy for globus pharyngeus between January 2005 and October 2008. Fifty-five patients were contacted by phone on average 5 years and 3 months after intervention and asked if their symptoms still persisted. Twenty-four patients were uncontactable or lost to followup, three patients were deceased, two of cardiac related disease and one of renal failure. *Results*. The majority of patients, 36 of 55 (65%), had a normal barium swallow. Forty-five of 55 (82%) of patients had normal rigid endoscopies. Thirty-one of 55 (56%) patients were at an average followup time of 5 years and 3 months. No patient developed a malignant lesion. *Conclusion*. Globus pharyngeus is a relatively common but benign condition of indeterminate origin. Our study demonstrates that many of these patients spontaneously improve with time.

## 1. Introduction

Globus Pharyngeus is a common disorder of indeterminate origin and constitutes about 5% of all new ENT referrals [1]. Patients commonly describe the sensation of a foreign body or tightness in the throat, and the literature reports a slight female preponderance [2]. It was first described by Purcell in 1707 who coined the term globus hystericus, the word globus originating from the Latin meaning “ball” and “hystericus” reflecting the then assumed psychological component of the disorder [3]. It was defined in the Oxford English Dictionary in 1794 as “a choking sensation, as of a lump in the throat, to which hysterical persons are subject,” and traditionally patients presenting with globus symptomatology were referred to psychiatrists. The disorder was renamed globus pharyngeus in 1968 [4]. Globus Pharyngeus has more recently been defined as (i) a persistent or intermittent sensation of a lump or foreign body in the throat for at least 12 weeks, (ii) occurrence of the sensation between meals, (iii) absence of dysphagia and odynophagia, (iv) absence of pathological gastroesophageal reflux (GERD), achalasia, or other motility disorder with a recognized pathological basis (e.g., scleroderma of the oesophagus) [5].

To date much has been published on the proposed aetiology of globus pharyngeus, which still remains poorly elucidated, and many theories have evolved. It has long been held that many of these patients have a psychogenic component to their disorder; however, from the mid-part of the last century the focus shifted to potential organic causes. In the 1940s cricopharyngeal spasm was a favored cause; however, manometric studies in later years found no supporting evidence [6]. Local lesions such as lingual tonsillar hypertrophy were postulated in the 1950s [7]. Cervical osteophytes [8] and iron deficiency anemia were proposed in the 1960s, but again the evidence was conflicting [9]. Perhaps the most controversial, and indeed most debated of potential causes, is GERD. The role of GERD was first postulated by Malcomson in 1968 who noted the presence of reflux on the barium swallows of over 60% of patients with globus pharyngeus; however, studies using 24-hour ambulatory pH monitoring of the lower oesophagus in subsequent years have produced conflicting results [4, 10]. Temporomandibular joint disorder was proposed in the 1980s but was never substantiated [11]. More recently the role of thyroid pathology has been explored [12]. While the exact aetiology remains elusive the current thinking is that globus pharyngeus most probably has a multifactorial origin [13].

Another drawback or difficulty in the management of globus pharyngeus is the lack of conformity when evaluating these patients. Investigation, workup, and indeed treatment of this group of patients vary from institution to institution. This was evidenced by a recent UK-based study which found that the favored mode of investigation amongst UK-based ENT consultants was rigid endoscopy performed by 61%, barium swallow was performed by 56% of consultants, 17.5% perform both barium swallow and rigid endoscopy. Fourteen and a half percent of consultants perform no investigations further to routine OPD evaluation [13]. The principal reason for investigating these patients is to out rule an aerodigestive tract malignancy. However, the majority of studies to date have failed to demonstrate a link between globus pharyngeus and the development of upper oesophageal malignancy yet many of these patients undergo at least a barium swallow and many, ultimately, rigid endoscopy. Furthermore, there is increasing evidence in the literature to suggest that many of these patients' symptoms improve progressively, and in many instances, completely resolve with time [14]. The longest followup study to date conducted by Rowley et al. in the 1990s found that over half of these patients were asymptomatic at 7 years and no patient developed malignancy during the study period [14]. While there are a wealth of studies in the literature examining the exact aetiology of this disorder, relatively few studies to date have addressed the value of barium swallow and endoscopy in this group. It is for this reason that we initiated this retrospective study, the principal aims of which were, firstly, to evaluate the role of barium swallow and endoscopy in these patients, to ascertain the incidence, if any of aerodigestive tract malignancy in this group and finally to assess the natural progression of globus pharyngeus.

## 2. Materials and Methods

This study retrospectively identified all patients over a 3 1/2-year period in our institution that had undergone oesophagoscopy for globus pharyngeus, where patients had experienced symptoms for at least six months. Data was obtained from clinical, operative, and radiological notes. All patients had a barium swallow prior to oesophagoscopy. Barium swallow involved a two-frame per second cinefluoroscopy of the pharynx and cervical oesophagus from the anterior-posterior and lateral position with additional single frames of the thoracic oesophagus. All patients underwent routine ENT examination, including indirect laryngoscopy and/or nasoendoscopy, and all had a normal video fluoroscopy. Exclusion criteria, which included use of barium swallow and oesophagoscopy, included patients with a history of pharyngeal and oesophageal malignancy or neurological disorders known to cause pharyngeal or oesophageal dysfunction. Patients had no prior therapeutic intervention for their symptoms. Data was obtained from the radiological and clinical notes on patients who underwent barium swallow and oesophagscopy during the study period. Patients were contacted by phone on average 5 years and 3 months (range 3 years–7 years 8 months) after intervention and asked if their symptoms still persisted.

## 3. Results

Fifty-five of 79 (69%) patients who initially underwent rigid endoscopy for globus pharyngeus between January 2005 to October 2008 were included in the study. Twenty-four of 79 (31%) of patients were uncontactable at the time of the study. Three patients died during the study period, 2 of cardiac related disorders and 1 of renal related disease. The majority of patients, 45 of 79 (57%), were female, 34 of 79 (43%) were male. The mean age of patients was 52.9 years (age range 23–79 yrs).

The majority of patients, 51 of 79 (65%), had a normal barium swallow. Benign lesions were detected on barium swallow in 28 of 79 (35%) of patients. The most common finding was cricopharyngeal spasm, detected in 9 of 79 (11%) of patients. Seven of 79 (9%) had a hiatus hernia, 7 of 79 (9%) had cervical osteophytes, and 5 of 79 (6%) had radiological evidence of reflux. Sixty-three of 79 (80%) patients had normal rigid endoscopies while benign abnormalities were detected in 16 of 79 (20%). Twelve of 79 (15%) had evidence of reflux, 3 of 79 (4%) had cricopharyngeal spasm, 1 of 79 (1%) had pharyngitis. A diagnosis of cricopharyngeal spasm was made on the presence of resistance to passage of a rigid endoscope at the level of the cricopharyngeus, where passage of the scope was variably achieved. When contacted by telephone on average 5 years and 3 months after intervention, 31 of 55 (56%) patients had symptomatic improvement 24 of 79 (44%) complained of persistent symptoms. Patients who improved with time complained of symptoms on average 13 months (range 3 months–3 yrs 7 months) before noting an improvement. No malignant lesion was detected on oesophagscopy or barium swallows and no patient developed a neoplastic lesion during the course of the study period.

## 4. Discussion

Consistent with much of the data in the literature there was a slight female preponderance in our series and the mean ages of patients were also consistent with published data. However, the wide range of ages reported by many authors was not observed in our study (range 17 years–79 years). Harar et al. reported a range of eleven to 96 years; however, this may be explained by the larger numbers in their study series [15]. 

No consensus has yet been established for the optimal evaluation of patients with globus pharyngeus. Isolated studies have demonstrated malignancies associated with globus pharyngeus; However, the majority of these patients had other “red-flag” symptomatology and thus did not strictly meet the criteria for a diagnosis of globus pharyngeus [15, 16]. The primary reason for investigating patients with globus pharyngeus is to rule out a neoplastic lesion. However, it is well established that barium swallow permits limited visualization of the postcricoid region and piriform fossa and therefore cannot definitively out rule a malignancy. Nonetheless, both barium swallow and rigid endoscopy are popular methods of investigating these patients as highlighted by Webb et al. [13]. Their postal questionnaire of UK-based ENT consultants showed that 86 percent of respondents perform investigations further to routine ENT evaluation. The preferred method of investigation was rigid endoscopy preformed by 61% of respondent, while 56% cited barium swallow as their preferred method of evaluation. Malcomson found that 63 percent of globus patients had a lesion on or at the level of the gastroesophageal junction on barium swallow [4]. However, Takwoingi et al. found that barium swallow had a limited role in the evaluation of the globus patient demonstrating the presence of a hiatus hernia and gastroesophageal reflux in 9% and 9.6% of patients [17]. Back reported reflux in 18.5 per cent (*n* = 92) and Batch had similar results [16, 18]. In our series, 6% had evidence of reflux on barium swallow (5 of 79) and 15% had evidence of reflux on endoscopy (12 of 79). Laryngopharyngeal reflux was diagnosed on rigid endoscopy by features such as vocal cord edema, diffuse laryngeal edema, the presence of thick endolaryngeal mucus, or posterior commissure hypertrophy. On this basis 12 of 79 (15%) were commenced on a course of proton pump inhibitors. Of these patients 7 of 12 (58%) noted complete resolution of their symptoms, 4 of 12 (33%) noted an improvement and only one (9%) patient reported no change in symptoms after a course of proton pump inhibitors. Numerous studies have investigated the potential role of acid reflux in globus pharyngeus including various combinations of barium swallow, 24 PH monitoring, oesophageal manometry, Bernstein acid test and flexible oesophagocsopy. Barium swallow, in contrast to the above investigations, is a much less sensitive investigation for acid and is associated with a high rate of false positive results. Cricopharyngeal spasm and cervical osteophytes have also been postulated as causes of globus pharyngeus; however, only 11% (9 of 79) and 9% (7 of 79) respectively, were noted in our series. The majority of cases of cricpharyngeal spasm result from the presence of a criocopharyngeal bar which may be treated by cricopharyngeal myotomy. While the numbers diagnosed radiologically with cricopharyngeal spasm were low, it did represent the most common abnormality detected in our study group. These figures are consistent with much of the data in the literature.

 Several studies have assessed the natural outcome of globus pharyngeus, mostly short-term outcomes. Wilson et al. found that 73% of their study sample were still symptomatic at 31 months [19]. The longest mean period followup study to date was conducted by Rowley et al. who found that 45% of patients had persistent symptoms at 7 years [14]. Our study showed that over half of patients symptoms had completely resolved at five years and 44% of patients were still symptomatic.

The benign nature of globus pharyngeus is again highlighted in a study by Caylakli et al. where most patients in their study series were found to have psychogenic morbidity and have no serious underlying pathology [20]. The possibility that there is at least a benefit to performing barium swallow and rigid endoscopies in terms of being able to reassure patients that they have no underlying malignancy has also been investigated. However, in our own series despite investigation and reassurance 44% of patients were still symptomatic at average followup of 5 years. 

The obvious pitfall of our study, and the majority of published studies in the literature on this subject, is its retrospective nature and thus dependence on clinical notes for details, relying on the history taking, clinical examination and documentations of each individual specialist. The same would apply for the barium swallows and rigid endoscopies preformed. Future studies should probably be designed as prospective cohorts.

It is still not clear if these patients require investigation, and, if so, what is the best method. The main indication for investigating these patients is to out rule a malignant lesion. However, there is no evidence in the literature to suggest that these patients ultimately develop upper aerodigestive tract malignancy [15]. Is it justifiable to expose these patients to the attendant risks of rigid endoscopy such as general anesthesia, dental trauma and perforation? We know the risk of perforation during diagnostic procedures is 1.2% [17]. The value of barium swallow is also questionable [18]. In addition, radiation exposure from barium swallows is significant with each barium swallow being the equivalent of 75 chest X-rays or 1.5 millisieverts of radiation [21].

Our results are consistent with much of the data in the literature to date demonstrating that many of these patients' symptoms progressively resolve with time. None of the patients in our study received any additional intervention after their initial endoscopy or barium swallow. The only treatment administered was in the form of a proton pump inhibitor. In addition, no patient in our study developed a malignant lesion in the pharynx or oesophagus. The most appropriate mode of investigation in this group remains unknown, but, in a recent paper, Takwoingi concluded that the role of both rigid endoscopy and barium swallow in these patients is limited and the present policy of endoscopically investigating all these patients is not evidence based [17]. 

The benefits of evaluation have to be weighed against the risk, expense, and the low probability of finding an abnormality that will ultimately affect treatment decisions, particularly in patients in whom the sensation has been present for many years. The current consensus in the literature is that if a diagnosis of globus pharyngeus can be confidently made on clinical grounds then reassurance is adequate. When there are other features associated with the clinical history, dysphagia, odynophagia, otalgia, fetor, or weight loss, then further investigations are mandatory.

## 5. Conclusion

 Our study results and a literature review support the existing evidence that a barium swallow and rigid endoscopy add little in terms of real clinical value in the evaluation of patients with globus pharyngeus. In patients where “red-flag” symptoms are present, interventions such as endoscopy are obviously indicated. If a diagnosis of globus pharyngeus can be made confidently on clinical grounds, then no investigation further to transnasal fibre-optic flexible endoscopy is required. Patients should be reassured of the benign nature of their condition and told that in many cases the condition resolves spontaneously. More invasive evaluation of these patients is not evidence based.

## 6. Summary

- Globus Pharyngeus is a benign disorder of indeterminate cause.
- No study to date has demonstrated that patients with globus pharyngeus ultimately develop upper aerodigestive tract malignancy.
- A recent study [17] demonstrated that rigid endoscopy and barium swallow add little valuable information in evaluating these patients.
- Our study is consistent with much of the data in the literature; the majority of barium swallows and rigid endoscopies in our series were normal, and no patient developed a mitotic lesion in the followup period.
- On the strength of our study and other recent publications the authors suggest that barium swallow and rigid endoscopy no longer be considered as first-line evaluation in patients with straightforward globus pharyngeus.
